# Interprofessional Collaboration between General Physicians and Emergency Department Teams in Belgium: A Qualitative Study

**DOI:** 10.5334/ijic.2520

**Published:** 2017-10-02

**Authors:** Marlène Karam, Sandra Tricas-Sauras, Elisabeth Darras, Jean Macq

**Affiliations:** 1 Catholic University of Louvain, BE

**Keywords:** interprofessional collaboration, integration, primary care, emergency department

## Abstract

This study aimed to assess interprofessional collaboration between general physicians and emergency departments in the French speaking regions of Belgium. Eight group interviews were conducted both in rural and urban areas, including in Brussels.

Findings showed that the relational components of collaboration, which are highly valued by individuals involved, comprise mutual acquaintanceship and trust, shared power and objectives. The organizational components of collaboration included out-of-hours services, role clarification, leadership and overall environment. Communication and patient’s role were also found to be key elements in enhancing or hindering collaboration across these two levels of care.

Relationships between general physicians and emergency departments’ teams were tightly linked to organizational factors and the general macro-environment. Health system regulation did not appear to play a significant role in promoting collaboration between actors. A better role clarification is needed in order to foster multidisciplinary team coordination for a more efficient patient management. Finally, economic power and private practice impeded interprofessional collaboration between the care teams.

In conclusion, many challenges need to be addressed for achievement of a better collaboration and more efficient integration. Not only should integration policies aim at reinforcing the role of general physicians as gatekeepers, also they should target patients’ awareness and empowerment.

## Introduction

The utilization of emergency department services in Belgium has significantly risen during the past few years [1]. In 2014, the National Institute for Health and Disability spent a total of 85.11 million Euros on the Belgian emergency services. This figure represented a 40% increase in expenditure compared to the previous five years [2].

Literature indicates that demographic and societal factors such as the rising number of older adults with increasingly complex health care needs, could account for this phenomenon [3]. Moreover, the fastest growth in emergency services utilization has been observed among patients aged above 65 years, especially those above 80 years [4]. This observation is particularly striking in Belgium where nearly 17% and 5% of the population is aged above 65 and 80 years respectively, against an overall average of 15% and 4% in countries comprising the Organization for Economic Cooperation and Development [5]. Up to 28.5% of the Belgian population above 15 years of age have reported suffering from at least one chronic disease in 2013 [6]. Yet, it has been demonstrated that patients with chronic illnesses use hospitals and emergency rooms at a higher rate than does the general population [7,8].

Organizational factors such as models of after-hours primary medical care services have been found to contribute to this situation [9,10,11]. Many European countries have recognized the need to reorganize out-of-hours services to meet patients’ high demands for primary care while maintaining quality and safety of care and satisfaction for both patients and health care professionals [12]. In Belgium, out-of-hours primary care services were initially organized by local general physician organizations, called general physician “circles”, via rotation systems for the residents of a specific geographical area. Since 2003, out-of-hours centres and walk-in clinics also known as “organized duty centres”, have started to develop, through public funding and on the initiative of general physician circles. They aimed both at rationalizing their on-call duties and decreasing home visits so as to improve working conditions [1]. Some of these centres are positioned in close proximity to hospital emergency services with the potential benefit of decreasing overcrowding of these services in two ways: firstly, by providing primary care for patients who do not have a general physician or whose physician is unavailable, thus decreasing unnecessary visits to emergency departments; secondly, by enabling emergency department to refer patients with primary care needs [13]. A single contact phone number (1733) has been made available in some regions in order to centralize patients’ out-of-hours calls and regulate them according to guidelines established by the general physicians [13].

The reorganization of out-of-hours services has led to conflicting results. In 2014, the National Institute for Health and Disability reported a total of 1.31 million emergency department visits registered at night, over weekends or during holidays– in other words, after-hours – against 507,999 visits in 2010, representing a 116.84% increase [2]. The Belgian Health Care Knowledge Centre is more confident in the potential benefit of organized duty centres and claims that their disappearance could negatively impact on emergency department activity. According to that Centre, the increase in after-hours visits is majorly accounted by the fact that the organized duty centres were initiated bottom-up without clear national guidance. Consequently, there is no obvious logic in how they are distributed across Belgium and there is a large variability in their operating approaches. For example, out of 70 organized duty centres in 2015, only seven were open during the evening on week days [1]. In parallel, the absence of a gatekeeper model allows patients free access to the emergency department of their choice at any time, without being referred by a general physician. Other organizational elements related to the Belgian healthcare system should be considered, including patients’ right to a second opinion in primary, secondary, and tertiary care services that brings about the utilization of more than one emergency department service for the same episode of care. Moreover, in the emergency department there is no direct payment of fees as opposed to visits to the general physician and out-of-hours services [14]. Emergency department utilization is known to be influenced by individual preferences such as the convenience of out-of-hours care [9], access to a full range of services at any time [15], the severity of illness as perceived by the patient and the confidence in their general physician’s ability [16]. In Belgium, this combination of factors accounted for the majority (70.3%) of self-reported emergency visits in 2012 [1].

In Belgium, healthcare services are provided by a combination of public and private sectors, funded by mandatory social security contributions proportional to house-hold-income, and characterized by universal coverage and equitable access to care [17]. Generally, patients receive a 75% refund of the cost of medical treatment or consultation and are responsible for paying the remaining 25% [18]. Medical primary care is often delivered by independent general physicians, working in the private sector, and paid on a fee-per-service basis. Another model of primary care delivery, still marginal, is through integrated health centres also known as Community Health Centres. Two models of payment are used in Community Health Centres, fee-per-service and the capitation fee in which the National Institute for Health and Disability Insurance monthly pays the centres a fixed contribution per patient registered with a sickness fund [19]. Community Health Centres enroll patients into their services based on geographical proximity, and provide them with preventive and curative care through multidisciplinary teams. In return, patients agree not to look elsewhere for primary care.

Another characteristic of the Belgian healthcare system is the limited attractiveness of general practice to young doctors [20], and the ageing active workforce. A recent report on working general physicians, covering the period between 2004 and 2012, showed that 62% were between 45 and 64 years old [21]. As these general physicians approach retirement age without sufficient younger recruits to replace them, the potential shortage of general physicians will become a major concern for the Belgian healthcare system. This situation is particularly alarming for emergency departments because reduced access to general practice has been associated with a higher utilization of emergency services [22,23].

The rise in emergency department utilization aforementioned poses a significant challenge in terms of continuity of care for patients regardless of them being referred by their general physician or not. It also justifies the assessment of interprofessional collaboration across primary and secondary care levels. Interprofessional collaboration in health care is a process by which professionals from different disciplines collaborate both to provide an integrated and cohesive approach to patient care [24], and to attain a more tailored and synchronized health care delivery [25]. This approach has been reported to decrease fragmentation of care and to increase the efficiency of healthcare systems by reducing redundant medical tests and associated costs, and even patient readmission [26].

However, interprofessional collaboration is a complex process, affected by a broad range of factors both internal and external to the health care team. Therefore, multidisciplinary teams vary in the extent and effectiveness of collaboration [27]. Interprofessional collaboration between general physicians and emergency department teams has not been sufficiently explored. Often, studies merely address communication between actors involved [28,29,30], which is just a component of interprofessional collaboration. The entire gap between primary and secondary health care levels remains incompletely unaddressed. This issue is particularly important in healthcare systems where few integration strategies, at an organizational level, are designed to enhance collaboration between actors.

### Aim and research questions

This paper explores experiences of collaboration between general physicians and emergency department teams in French-speaking regions of Belgium. It aims at identifying the main concepts related to interprofessional collaboration and highly valued by involved actors. We focus on positive experiences of collaboration. More specifically, we sought to find out what works between general physicians and emergency department teams in terms of interprofessional collaboration? And in which contexts and conditions?

### Theoretical framework

D’Amour’s theoretical framework of interprofessional collaboration considers aspects of structure and relationships between individuals. Organizational dimensions include formalization and governance, whereas relational dimensions include shared goals and vision among healthcare professionals and internalization. The model also focuses on the interaction between the organizational dimensions and inter-individual relationships [31]. It also has the strength of being derived from theoretical and empirical data and having been tested and validated by its authors. Hence, we considered D’Amour’s framework as particularly suitable for our study.

## Methods

### Appreciative Inquiry

The concept of focusing on positive experiences draws on the Appreciative Inquiry philosophy, which is based on the assumption that every organization has something that works well and these strengths can be the starting point to create positive change [32]. The concept consists of helping participants to recognize and value what works best in their organization, rather than other traditional approaches focusing on difficulties and problems.

### Design and settings

We used a qualitative approach to explore experiences of interprofessional collaboration between general physicians and emergency department teams. A series of eight group interviews with both groups were conducted in Brussels and other areas of French-speaking Belgium. Given the unavailability of research addressing this topic, we carried out an exploratory study without pairing of study areas. However, through purposive sampling, we assessed experiences in a variety of settings, that is, both in rural and urban areas including in Brussels.

In Belgium, general physicians have to belong to a peer review group in order to keep their accreditation. Peer review groups are composed of eight to twenty-five general physicians who meet four times a year to discuss specific themes related to their practice. The groups are chaired by a “rapporteur” peer who is responsible for organizing the meetings and keeping the list of participants up to date.

Eighteen rapporteurs both form rural and urban areas were contacted by email. They were requested to include our study in their meetings. Five groups agreed to participate in the interviews, two from Brussels, one from the province of Namur where general physicians cover both urban and rural areas, and one from Baudour which is an urban area. However, only four of the five group interviews took place; the fifth was cancelled because of scheduling conflicts. Two rapporteurs declined because the discussion topics for their meetings were set several months in advance. We had no replies from the other eleven groups.

We asked each rapporteur to inform their group in advance about the topic of discussion, and to stress the nature of the research, that is, to discuss positive experiences of collaboration with emergency department teams.

At the same time, we recruited emergency department teams with the same aim of covering diversity of settings. We contacted, again by email and telephone, medical and nursing directors and heads of emergency departments of nine hospitals from different areas. Four hospitals agreed to participate, one from Brussels, one from Dinant which is a rural area in the province of Namur. The remaining hospitals were from Ottignies and Baudour, two urban areas with significant socio-demographic differences (see Table 1). One hospital refused to participate because of unavailability of the team. Four hospitals did not answer despite a reminder.

**Table 1:** Demographic and socio-economic characteristics of the study areas.

Area	Setting	Province	GPs’ density/10 000 population (per province) [33]	Number of EDs per province [1]	Number of EDs per area [1]	Out-of-hours services	Socio-economic characteristics

Brussels	Urban	Brussels	15.1	19	19	ODC and central phone number	Diversity in origin, cultural background and socio-economic status. About one third of the population is living with an income below the risk threshold of poverty. 82.7% of the population have a regular GP [34]. In average, they consult their GP 2,8 times per year [35].
Ottignies	Urban	Walloon Brabant	17.9	4	1	Absence of ODC and central phone number	Good socio economic status in general, and a higher average income in comparison with Wallonia (+23,3%) [36]. High population growth since 1990 (+23,2%) with increased educational level [37].
Dinant	Rural	Namur	17.6	6	2	ODC and central phone number	The socio-economic indicators (average income, unemployment and social integration income) are less positive compared with the province of Namur [38]. 95% of the population have a regular GP. No difference is noted between urban and rural areas [39].
Namur	Urban/rural				4		A better socio economic situation than Wallonia in general. Growing and aging population (+14,6% and +29,1% since 1990) [40].
Baudour	Urban				8		Low socio economic status, lower incomes compared to the Belgian population, more unemployment, more single-parent families, fewer tertiary graduates [41]. 95% of the population have a regular GP. They consult their GP 4,4 times per year in average [35].
Gosselies	Urban	Hainaut	12.7	19		ODC and central phone number	
					6		

**Legend:** GPs refers to General Physicians. EDs refers to Emergency Departments. ODC refers to Organized Duty Centers. Calculation of EDs number for Baudour includes three areas: Soignies, La Louviere, Mons.

We agreed on the date and time for the group interviews with each head of department according to their preferences. In line with our study objectives, we requested them to invite different members of the multidisciplinary team, including social workers, secretaries, nurses and emergency medicine doctors to the discussion. Again we asked that the participants were informed in advance about the topic of discussion.

Our sample included 65 participants: 35 general physicians, 24 emergency doctors, 4 nurses, 1 social worker and 1 secretary (see Table 2). The practice setting of the general physicians and emergency department informants are reported in Table 3.

**Table 2:** Description of data collection and participants’ characteristics (N = 65).

	GROUPS								
	Sequence of interviews	GI 1	GI 2	GI 3	GI 4	GI 5	GI 6	GI 7	GI 8
	Context	Brussels	Brussels	Baudour	Dinant	Namur	Ottignies	Gosselies	Brussels
	Investigators	MK and ST	MK and ED	MK and ST	MK and ST	MK and ST	MK and JM	MK and JM	MK and JM

**Profession**									
General physician		9	7	6		13			
Emergency physician					9		5	4	6
Emergency nurse					1		1	1	1
Social worker					1				
Secretary					1				

**Legend:** GI refers to Group Interview. MK, ST, ED and JM refer to authors/investigators initials. **Used in verbatims:** Emergency Physician: EP. General Physician: GP.

**Table 3:** Practice settings of the participating general physicians and the emergency departments they work with.

Area	Practice setting	Collaborating emergency departments

Brussels	Private practice/ community health centres/ Screening centres/ mental health centers/ polyclinics	Private and public hospitals within Brussels according to the patient socio-economic characteristics, preferences and geographical proximity
Brussels	Private practice/ community health centres/ family planning centres/ polyclinics	
Namur	Private practice/ community health centres/ polyclinics	Hospitals within the province of Namur, Dinant, Mont-Godinne, and Ottignies
Baudour	Private practice	Hospitals within the Hainaut: Baudour, Tivoli, Jolimont, Soignies, and Ambroise Paré

### Ethical considerations

Ethical approval was obtained from the Hospital and Departmental Ethics Committee, Saint-Luc – Catholic University of Louvain, in Brussels. Additionally, prior to each interview, the first author was reminding participants about the objective and nature of the study. Only after that could the written informed consent be requested.

### Data collection

Group interviews were conducted between September 2014 and December 2015 depending on participants’ preferences and availability. The sequence of the interviews is shown in Table 2. Interviews of general physicians took place during the scheduled meetings of their peer review group, in a quiet room, late in the evening at the end of their consultation. For the emergency teams, group interviews were scheduled early in the morning, before the patient “rush hour”, in a relatively isolated room in the emergency department. Only one emergency team interview took place late in the evening, in one of the hospital’s meeting rooms, away from the department. The first author was present at each meeting with another investigator; one of them could moderate the group discussion while the other was taking hand-written notes (see Table 2). Participants were informed about the backgrounds and current occupation of each investigator.

The first author is a senior PhD candidate, with expertise in qualitative research acquired through both research and teaching activities. The three other authors are senior researchers and have extensive experience in qualitative and health systems research. The first author has a background in emergency nursing and has previously worked with some of the participants from different emergency departments, which facilitated the storytelling within certain groups. However, her background did not impede interactions within the general physician groups since it was made clear that investigators’ role was non-judgmental; and they would be merely interested in what they were being told. The interviews lasted about one and a half hours on average. They were audio-recorded and transcribed verbatim.

A topic guide rather than a predetermined list of questions was used to explore experiences. It consisted of 4 to 5 open-ended questions with follow-up prompts used to generate further discussion (see appendix 1). The guide evolved during the data collection period as interviews produced new information about various aspects of experiences of collaboration. It was based on the first two phases of the Appreciative Inquiry: “Discovery” during which data are gathered in the form of positive stories of interprofessional collaboration, and “Dream” in which participants are requested to co-create a shared vision of a preferred future [32].

### Data analysis

Rapporteurs and heads of departments provided the general physicians and emergency department teams with summaries of their group discussion for validation, respectively. The summary was validated for only three groups; though in our email it was specified that, for the convenience of the participants, the absence of any feedback from them within two to three weeks would be considered as an approval of the summary.

An iterative approach was used. At the end of each meeting, the two investigators met and discussed the collected data. Field notes allowed a preliminary crossed analysis which revealed emergent themes after each group interview. The crossed analysis also helped minimize potential interpretation bias that might have arisen from the first author’s professional background.

At the end of the eighth group discussions, we were considering that descriptive saturation was reached since new themes were no longer emerging, although within identified themes different stories and experiences could continue to be shared.

Transcripts were analysed thematically using an integrated approach that employs an inductive development of codes and a deductive organizing framework for code types [42]. We used a start list of two predefined categories: relational and organizational components of collaboration, in accordance with D’Amour’s structuration model. The first and last authors independently coded the transcripts and identified emergent codes through repeated examination. We then compared, combined and organized these emergent codes within the two predefined categories.

Nvivo 10 software was used for texts organization and management.

## Results

The results are presented with respect to relational and organizational components of collaboration. In addition, two independent categories emerged from the thematic analysis: communication, whether written, oral or electronic; and the patient’s role in enhancing or hindering interprofessional collaboration.

### Relational components of a positive experience of interprofessional collaboration between general physicians and emergency department teams

#### Mutual acquaintanceship and trust

Mutual acquaintanceship was found to be a major component of a positive experience of collaboration. Both parties agreed that knowing each other optimizes contact and enhances relationships. When asked to “dream” about a desirable future of collaboration, participants raised mutual acquaintanceship as a common priority:

“Meeting each other opens doors, facilitates communication and breaks the anonymity” (GI 5).

“When we know each other, the contact is completely different” (GI 4- EP).

This mutual acquaintanceship is seldom the result of formal meetings such as seminars or scientific reunions. Both parties admitted not organizing and rarely attending such activities because of the heavy work load, lack of time and funding. At the same time, both general physicians and emergency teams were aware of a void in this area and would like to meet more frequently and know of each other. Participants reported that they knew each other on a more informal basis. They either worked together for long periods, or were classmates at medical school, or had met during pre-hospital emergency intervention or a festive occasion.

Trust seemed to emerge as a result of this mutual acquaintanceship. Both groups said that trust develops over time when they share experiences that cause them to rely on each other. For participants, trust is manifested in seeking advice from each other and making joint decisions, which in turn create a sustainable and strong relationship:

“Since that moment, I feel like something has changed, ties have been strengthened” (GI 1).

Inevitably, negative experiences also have an impact on trust. In this case, mutual acquaintanceship becomes irrelevant:

“There are some we know, but can hardly trust” (GI 7- EP).

Finally, trust is also dependent on the perception of hidden intentions, especially when it comes to “stealing” the patients from others, within a healthcare system that allows such a competition. For example, some emergency team members are still wary of the physicians’ main objective for installing organized duty centres in front of the hospital entrance.

“They actually wanted to recruit patients and stop them from going to the emergency department” (GI 4- EP).

#### Power

A positive experience of power was, for both sides, characterized by a relationship of equality without dominance behavior. It is demonstrated by respectful and professional attitudes towards each other, mainly in terms of verbal communication:

“… I mean, when we have esteem and consideration for each other and can communicate as equals…” (GI 4- EP)

“… Because there is a world, a certain hierarchy where emergency physicians and specialists are on top, and general physicians at the bottom…usually it’s on a case-by-case basis, but we see that in lots of hospitals…” (GI 3).

Shared power is thought to be strongly linked to involving others in the decision-making process:

“It’s about not excluding me from participating in my patient diagnostic process and plan of care” (GI 2).

Both parties thought that emergency department teams had a certain degree of power as a result of much more technical resources they have at their disposal, the relatively easy access to medical imaging, and the availability of lab results in a short timeframe. Some of the emergency teams were considering their scientific knowledge as a source of power whereas general physicians were conflicting this opinion, arguing that this form of power was only related to the better technical resources of the department:

“If they had the same technical means that we have, I am not sure they would know better than we do” (GI 1).

As for emergency department teams, they considered that general physicians exert their power through the privileged relationship with their patients by two means. Firstly, it is by communicating to the patient their presumed power over the emergency department team:

“They tell the patient “*I’m referring you to the emergency department where you ARE going to have such and such exams, I have requested them in my note, so they HAVE to do it*” (GI 4- EP).

This type of information creates expectations that influence patients when demanding medical exams and can lead to conflict. It can also create confusion of the emergency department with a technical platform.

Secondly, when competition between hospitals is intense as a result of close proximity, general physicians hold the economic power as they are the “patient providers”. The hospital in general becomes dependent on their referrals. Thus, the emergency department team becomes trapped between the direct demands of the general physician and the institutional policies aimed at satisfying these demands. This situation is not observed in Brussels where most patients do not even have a general physician, or move from one hospital to another without informing their physician.

Finally, some of the interviewees deemed the term “power” inappropriate. For them it was more about moments of tension because of local conditions. It would therefore be more appropriate to use terms such as consideration and sharing. For these physicians, power conflicts should not exist since the two groups don’t perform the same work:

“Everyone has got their own job, their own skills and powers” (GI 2).

#### Shared objectives

Despite perceived differences in professional cultures and practices, the interviewees agreed that they share one main objective, the patient’s wellbeing. Indeed, general physicians and emergency department teams come from different organizations with different management structures, various types of leadership and guidance as well as divergent priorities and objectives.

In parallel with this separation, both parties felt they were in a continuum whereby general physicians aim to maintain the patient’s wellbeing and health in their daily life when emergency department teams intervene on a more limited basis for acute episodes.

### Organizational components of a positive experience of interprofessional collaboration between general physicians and emergency department teams

#### Out-of-hours services

Experiences related to organized duty centres were mainly positive for both parties. These centres rationalize the on-call duties for general physicians and help decrease emergency department overcrowding. However, the relevance of integrating duty centres within the emergency services is debated. Some emergency department participants argue that there are many benefits of having “intramural” general physicians. It would facilitate referral to the general physician- who is just next door- and ease the orientation of the patient towards the emergency service. It would also facilitate case discussions and requests for an opinion among doctors, and enhance relationships. Additionally, emergency department participants think that integrated duty centres could offer a solution to the request made by general physicians for a “specific pathway” for patients whom they have already seen so as to avoid them from waiting to be seen again by the emergency department doctor.

The general physicians were formally opposed to this proposal and considered that general practice should remain a non-hospital and community-based practice. They recalled that the rationale for creating these centres was to organize the primary care out-of-hours services, but not to relieve the emergency departments.

#### Clarification of professional roles

Knowing one’s own role and others’, and fulfilling that role are considered valuable aids for collaboration for two main reasons. Firstly, these aspects assume that everyone is aware of and confident in other’s competence. Confidence brings about greater complementarity between roles of different actors:

“Each of us has learned different skills and, to me, a positive experience is when everyone brings their own skills and knowledge to the table” (GI 5).

Some general physicians do not hesitate to seek advice from the emergency department. They also emphasized that knowing that they can rely on the emergency teams to take over was a relief from the major stress inherent in critical emergencies.

Similarly, emergency teams often rely on the general physician’s input to better understand a patient’s psychosocial context, health and living conditions in order to guide the decision-making process and the patient discharge plan:

“A positive experience is when they help us with the decision-orientation, especially for elderly and chronic patients: discharge from hospital? Nursing home? Palliative care? Intensive care?” (GI 4- EP).

In line with the need for input from the general physicians and their role in decision-making, emergency teams “dreamed” of having end-of-life projects discussed in advance with the patient’s family, and a written report available in the patient’s record. This was seen as being valuable for both pre-hospital interventions in a nursing home and for in-hospital emergencies:

“We don’t know patients, and when we see them in emergency department or at home, of course they don’t look good, but it is difficult for us to know whether this is how they usually are, or if it is something more acute. This is something that the general physician could tell us… we start wondering which measures to take “should I intubate? Should I resuscitate?” (GI 6- EP).

Secondly, role clarification is thought to improve the referral process, thus decrease tensions between the different levels of care. For example, some general physicians have requested emergency teams to refer their patients back to them for removal of stiches instead of referring them to specialists. To them, this is a minor intervention and part of their competence.

Finally, some general physicians described their respective roles as existing in parallel, with nothing more than written patient reports as a communication tool, and thought this relationship was “enough” to ensure continuity of care.

#### Leadership

A positive experience of collaboration was also found to be linked to the presence of leaders who appreciate and value the expertise of others. For example, some heads of emergency departments invite their staff to understand the reality of general practice and its environmental constraints. They also instill a certain culture of respect, especially among newly graduated doctors:

“We have to realize that the general physician faces the challenge of achieving a balance between granting their patient what they request- sick leave, medical exams-, and remaining consistent and providing an adequate medical practice… No, they do not refer patients for anything and everything, behind their demand we have to realize the pressure of the family, the patient himself, the lack of material, etc.” (GI 6- EP).

Moreover, such leaders seem to play a significant role in balancing power by reassuring their teams that they do not need to abide by the requests of the general physicians for medical exams, but should rely on their own clinical assessment.

#### Environment

Experiences related to the impact of the environment on collaboration were rarely identified as positive. Results showed that some constraints are specific to the general physicians’ environment, others to the emergency departments. Moreover, both parties stated that the macro-environment does not play a significant role in enhancing collaboration among them.

The environmental constraints for general physicians are mainly the crowded waiting rooms and lack of time that prevent them from calling or writing a detailed letter for the emergency teams, or attending meetings or seminars with them. The lack of material and human resources also causes them to refer worrying patients to the emergency services before the weekend when facilities and medical cover are sparser. These referrals are not well considered by the emergency department teams. To them, general physicians should be more organized for after-hours coverage and more available during weekends instead of cluttering emergency departments every Friday evening with patients with primary care or social needs. Moreover, poor equipment of out-of-hours primary care centres is a major problem for on-call general physicians who see no other alternative than sending their patients to the emergency department for minor interventions.

The environmental constraints of emergency department teams can be summarized by the intermediate position of the department in between primary care and hospital units, that is, between general physicians and specialists. Several emergency physicians expressed the feeling of being caught between these two groups:

“What they (general physicians) don’t understand is that the decision of allocating a bed (hospitalization) is not ours, it’s the specialists… They sense our aggressiveness on phone when the hospital is full, the emergency service is full, and they call to request a bed for their patient” (GI 6- EP).

In parallel, general physicians said that as a result of this unwelcoming attitude, they stopped calling to inform of their referrals and chose to simply send patients to the emergency department by their own means.

From a more positive perspective, both parties are aware of their lack of knowledge about the environment and operating mode of others, and “dreamed” about a better mutual understanding of the other’s reality:

“A good thing would be to have this type of meeting with the emergency teams where they explain to us how the system works and how they operate” (GI 5).

General physicians put special emphasis on the need for exchanges by residents beyond the initial training period so as to expose them to the reality of general practice and help them understand it better, thus preparing the field for better future collaboration.

Finally, when questioned about the role played by the macro environment and healthcare system in general in collaboration enhancement between these groups, participants agreed that this role is minor since neither incentives-based measures nor clear strategies are used to bring them together:

“Nothing has been done to bring us closer, nothing, zero!” (GI 5).

### Communication

Communication was considered highly significant by participants and found to play a central role in collaboration. It encompasses both relational and organizational aspects of collaboration. Communication also involves informal and formal characteristics and tools, varying from friendly and conversational phone calls to a more structured type of communication such as shared electronic patient records.

#### Written reports

Positive experiences of collaboration were related to good communication. Both parties attached great importance to comprehensive, clear, timely and accurate written reports.

General physicians think that reports enhance the patient-carer relationship. To them, patients have more confidence in their physician when the latter knows about their visit to the emergency department or has a full report of the episode and thus is able to reinforce what has been said in the emergency room. However, despite remarkable progress in this domain, many written reports from the emergency department continue to arrive months after the health event and some are sent to inaccurate addresses.

Similarly, emergency department teams said they were pleased when patients referred to them by the general physician were accompanied by a clear report specifying the patient’s main complaint, their medical history and current treatment as well as a differential diagnosis. In parallel, unjustified requests by general physicians for medical exams were considered as inappropriate use of the emergency department:

“If the goal is to get medical exams, he might as well refer his patient directly to the radiology department or to the laboratory” (GI 7- EP).

“We really have the impression that they are confusing the emergency department with a technical platform” (GI 4- EP).

#### Oral communication

Phone calls are highly valued, largely because they allow direct interaction. For the emergency department teams, the general physician’s phone call guides them in the plan of care, even more than a written note, especially when referring a patient with a difficult socio-economic context. In this case, oral communication allows negotiation of the patient’s final destination and a joint decision-making process:

“It (a phone call) answers questions such as should the patient be kept hospitalized despite medical clearance? Do they have informal caregivers? What kind of help would they need if they were to be discharged, etc.” (GI 6- EP).

For general physicians, a phone call from the emergency department looking for this information is perceived as a sign of respect:

“We appreciate this type of call because we are obviously better placed to answer these questions” (GI 5).

Some general physicians reported that their calls to the emergency department for advice were well received. Their phone call represented a positive experience for them, thanks to its associated good interaction and efficacy, it helped them keep their patient at home and prevented a hospital referral.

On the other hand, some emergency doctors reported having received phone calls from general physicians who admitted being overwhelmed by patient or family pressure. They honestly acknowledged that in such a situation, they were finding themselves compelled to refer the patient to the emergency department, albeit unconvinced of the medical necessity of this referral. This was reported as a positive experience because of two major components of communication that are transparency and openness. Moreover, participants thought that the presence of these components was dependent on mutual acquaintanceship.

Despite the perceived benefit of oral communication, both parties are aware that it takes much more time to make a phone call than to write a note. The institutional constraints of the emergency department, the work load and the limited availability of the general physician make it increasingly difficult to call or to meet with each other. Participants thought that “hotlines” or direct access to a colleague without intermediaries may be part of the solution to this problem.

#### Electronic communication

Electronic communication is becoming a substitute for phone calls. Emails and medical software allow patient information to be rapidly and easily shared. Regarding shared patient records, known in Belgium as eHealth, participants thought it would become a very practical tool, although yet fully developed. Some participants from both sides stated that the development of an effective shared electronic patient record was one of their “dreams” of preferred future collaboration.

The main reported concerns about the current state and utilization of eHealth are related to ethical issues such as patient confidentiality or potential legal problems in case of medical errors. Enrolling patients after obtaining signed informed consents from them is also deemed to be a complicated procedure. More technical problems were also reported. For example, the general physician computer software is not licensed and therefore not compatible with the eHealth platform. Participants found it difficult to keep the platform up to date with patient information; they could also be reluctant to take on additional administrative tasks. In addition, some of the participants were either unaware of the existence of eHealth or unskilled at it.

Finally, the patient is perceived as the main beneficiary of communication, mainly due to the continuity of care, but also because it improves their experience of care.

“It reassures him to be expected, to be taken seriously, and to know that his caregivers are discussing his condition and agree upon his care plan” (GI 2).

Both actors “dreamed” of the presence of systematic bidirectional communication and availability of the other party as top priorities to achieving better future collaboration.

### Patient’s role in enhancing or restraining interprofessional collaboration

The patient is thought to be both a key component and the driving force of collaboration between the two levels of care:

“…Whenever we receive a phone call from the emergency department, we have this clear feeling that it is the patient’s explicit demand” (GI 1).

General physicians linked this demand to their privileged relationship with the patient and their greater trust in them than in emergency teams they see for the first time. They also viewed their request as a means of balancing the decision-making power between the two groups:

“It is as if he is saying: it is not up to you alone to decide, it’s also up to my physician” (GI 1).

To them, emergency teams recognized this privileged relationship and the trust between general physician and a patient, and at times they could use it to help involve the patient in the proposed plan of care.

In parallel with their role in enhancing collaboration between the two groups, patients may also create some barriers. Not only can they influence the referral decision, but some patients turn up at the emergency department on their own initiative, pretending that their physician wanted them to undergo specific medical exams or to be hospitalized.

## Discussion

The principal contribution of this study stems from the fact that interprofessional collaboration between general physicians and emergency department teams was so far scantily explored. To our knowledge, this study is the first to address issues of collaboration between these groups. We believe that scientific literature on collaboration in this setting should be developed further for two main reasons. Firstly, literature shows that the urgency of clinical work to be achieved has a major impact on collaboration. Urgent and acute medical crises affect team interactions in a number of ways, making teams work closely to deliver appropriate urgent care, or provoking brusque forms of interaction and divergent perceptions among the professionals [43]. Secondly, emergency department teams are constantly dealing with chronic patients presenting with acute episodes and need for medical care. In these cases, prompt and effective interprofessional collaboration is vital for reduction of errors [25,44] and helps ensure a continuity of care for patients across the two levels [45].

Another contribution concerns the central role played by the patient in enhancing or impeding efforts towards collaboration. Scientific literature has so far focused on interprofessional collaboration as a process that places the patient at the centre of care, supports shared decision-making and takes into account their own preferences and individual goals [25,46,47,48]. Sharing power with the patient has also been developed and questioned by Fox and Reeves [48]. Additionally, outcomes of collaboration have been measured at patient level, focusing on patient satisfaction, quality of care and safety [49,50]. This study sheds light on the impact of patients’ attitudes, behaviors and individual goals on collaboration between healthcare professionals. Our findings suggest that patients can be the driving force in collaboration by explicitly requesting healthcare professionals to communicate. On the other hand, they can impede efforts towards collaboration by transmitting incorrect or misleading information among healthcare providers.

### Summary of findings

D’Amour’s structuration model of interprofessional collaboration helped us throw new light on relationships between healthcare providers. It was also instrumental in helping us investigate the interaction between the relationships and organizational dimensions across the two levels of care. We showed that despite many positive experiences of collaboration, conflicting issues also emerged and were often linked to organizational dimensions.

Indeed, the way healthcare system is regulated in general did not seem to play a significant role in promoting collaboration between the two parties. The new legislation on hospital financing seems blurred and raises concerns and mistrust. The data and information exchange system remains poorly developed. There is neither gatekeeping process nor incentive measures to encourage collaboration between the two levels of care, on top of insufficient associated integration policies. However, a federal plan has been launched recently, aiming at developing integrated and patient-centred care for chronic conditions by developing pilot projects that trial and evaluate models of integration [51]. This plan is based on the triple aim principle for integrated care [52], and consists of 18 components including seamless care, coordination, continuity of care, shared electronic patient records, multidisciplinary guidelines, adaptation of the funding systems, continuing education on integrated care, and interprofessional education among others. Inevitably, pilot projects need to include both primary and secondary care actors. We expect that emergency services will also be involved because, in many cases, they are considered as the gateway to secondary care.

We also showed that there is a need for better professional role clarification. On one hand, communication and collaboration between the levels of care occur mainly between doctors. However nurses and social workers play a significant role both in organizing patient discharge and transition to home care and ensuring that health and social care needs are met. Involving the two disciplines in interprofessional interventions has been associated with positive outcomes such as enhanced multidisciplinary team coordination, involvement of family members in care planning and interventions, and enhanced quality of care for chronic disease management [49].

On the other hand, a minority of general physicians did not perceive any benefit of investing in collaboration. They thought that parallel practice was sufficient to ensure continuity of care. Both recognition and respect of the complementarity of roles can optimize professional scopes of practice, thereby ensuring more efficient patient management [53]. The role clarification process is not only an organizational issue or the responsibility of legislation alone, it is also up to individuals to clearly communicate all aspects of their different roles. Moreover, our study revealed a fundamental misunderstanding about the other group’s environment and operating mode. Improved communication could be the key to avoiding unnecessary tensions and improving referral processes.

Communication would allow both role negotiation and clarification [54,55] and foster relationships between individuals [56]. Efficient flow of information between teams would build trust up and allow ideas and decisions to be rigorously debated [57].

Finally, our findings regarding the patient’s role in enhancing or hindering collaboration raise the question of the degree of patients’ freedom of choice and the non-obligation of having a regular general physician. This study supports the fact that the inappropriate utilization of emergency departments is not systematically related to the unavailability of the general physician.

Experiences of interprofessional collaboration seemed to be homogeneous despite the heterogeneity of settings, with two exceptions. Firstly, in the context where the general physician is actually the “patient’s provider”, collaboration seemed to suffer from a power struggle and imbalance, at least from the emergency department team’s point of view. In large cities, this relationship of dominance does not exist because the general physician does not exert the same influence on a patient’s decision to visit a specific emergency department. This finding may appear paradoxical when considering the fact that there is a high density of hospitals in large cities, moreover in close proximity to each other. But again, patients’ freedom of choice may increase self-referral visits to any hospital and sometimes to several hospitals for the same health complaint.

Secondly, in one group of general physicians were not convinced of the necessity for collaboration, and believed that parallel practice was sufficient to ensure continuity of care for their patients. This group was composed of general physicians exclusively practicing in private, thus not being part of a multidisciplinary team at primary health care level like in community health centres or family planning centres. Working solo is most likely the main factor accounting for such statements. Skill-mix at primary health care level is believed to exert a major influence on the development of interprofessional collaboration [58]. It would enhance role clarification, involve coordination, and allow interdependent work and collective problem-solving across primary and secondary care levels.

### Recommendations

Appreciative Inquiry is designed to shift the focus from a problem-based research paradigm to a positive theory of inquiry based on future possibilities and performance [59]. Recalling satisfying experiences of collaboration, helped produce ideas and proposals for improvement based on existing strengths. However, participants also had some negative experiences to share. The need to discuss conflicting issues was expected. In fact, most of the participants emphasized that they had divergent professional and organizational cultures. In order to achieve better collaboration between actors at different health care levels in French-speaking Belgium, the following recommendations arose from both positive and negative experiences:

- Open, bidirectional and systematic communication is crucial to achieving trust and mutual acquaintanceship, thus fostering collaboration between all involved actors. In fact, dialogue is one of the main forces driving the implementation of collaborative care [60]; it can be achieved despite barriers like insufficient time and funding. For example, actors could take advantage of their scheduled meetings to invite the other party and discuss issues related to collaboration, namely role clarification, mode of functioning, organized duty centres, future legislation on hospital financing, etc. Also, informal meetings or team-building activities could be organized occasionally and would allow better interaction with less professional tension. Some participants suggested the use of hotlines for oral communication. Others asked for training basic information technology to enable better utilization of eHealth.
- To address economic power issues related to “patient provision”, participants argued that the trust of general physicians in emergency teams increases their loyalty to the hospital. Trust ought to be achieved gradually, by more positive experiences of collaboration and transparent communication. Meanwhile, raising patient’s awareness of inappropriate use of emergency departments appears urgent. There is also need for each patient to be having a regular general physician and a form of gatekeeper system.
- Interprofessional education has proved critical to enhanced collaboration [61,62]. In Belgium, several universities and nursing schools have recognized the need for an integrated approach to health professional education. They also took the initiative of organizing inteprofessional seminars and case studies. This gradual development of interprofessional education programs is still marginal and should be encouraged, promoted and financed by universities and the government.

### Limitations

Limitations of this study include the fact that we did not gather together both parties in the group interviews. Organizational constraints such as time and space impeded a possible arrangement of joint meetings with “the whole system is in the same room”. However, it is planned to gather the professionals together in a subsequent phase to disseminate the findings and seek feedback from them. Moreover, this study will be integrated into a supplementary phase that is assessing patients’ perception of continuity of care between their general physician and the emergency departments that participated in the group interviews. Lack of time and funding limited the recruitment process as well as participation of the target population. Both parties willingly participated without any compensation. Finally, participation of heads of emergency departments may have influenced the group dynamics, but « natural group interviews », akin to those conducted in this study, “are designed to provide a more “naturalistic” setting, resembling in some ways the kinds of interaction people might have in their daily lives” [63], pp 112.

## Conclusion

Both positive and negative experiences helped in understanding interprofessional collaboration between general physicians and emergency service teams. Many challenges remain before better collaboration and more efficient integration can be achieved. It appears essential that integration policies should reinforce the role of the general physician as a gatekeeper and target patient awareness and empowerment. Raising patients’ awareness of the necessity of having a regular physician is crucial, especially in the Belgian context.

## Additional File

The additional file for this article can be found as follows:

10.5334/ijic.2520.s1 Appendix 1 Topic guide.
