# Supplementary material for: Interprofessional Collaboration between General Physicians and Emergency Department Teams in Belgium: A Qualitative Study
Source: Int J Integr Care. 2017 Oct 2;17(4):9. doi: 10.5334/ijic.2520 (PMC5853879; doi:10.5334/ijic.2520)
Supplement: Appendix 1 — Topic guide. [file ijic-17-4-2520-s1.pdf]

Appendix 1: Topic guide (group interview with emergency department teams; slight adaptations were made for interviews with general physicians).

Warm up and establishing rapport:

- Thanking everyone for participating in the study;
- Informing participants about the names, backgrounds and current occupation of each investigator;
- Explaining the objectives of the study, its process and breadth, etc.
- Explaining how confidentiality and anonymity will be protected;
- Going over consent form and passing it to participants;
- Asking for permission to record and informing the participants that they can request to stop the recording at any time of the interview;
- Informing about the duration of the interview;
- Allowing participants to ask any question before the start of discussions;
- Inviting participants to introduce themselves.

1. Let's talk about situations where you find yourself working closely with general physicians.
   Prompts: What are these situations? At what moments? For which type of patients? ...

2. Think of a moment when your collaboration with a general physician seemed to be positive. Can you tell us this story?
   Prompts: What is it in this experience that "delighted" or pleased you?
   Can we say it was "…"? (e.g., shared power, trust, communication …); could you please explain your point?...
   How did this experience change your relationship with this general physician?
   What lessons can be learned from this experience?

3. Let's go back to "…" (i.e., communication); what is it that makes communication so important for you?
   Prompts: what type of communication? Tools? Formal, informal reunions? Who's the initiator? Whose job is it to facilitate communication? ...

4. Can you tell us about initiatives/policies/strategies, on a federal level, that enhance your relationships with general physicians; can you give some examples?
   Prompts: was it beneficial? How?

5. Imagine that you have a magic wand and that you are allowed three wishes or dreams that would help improve your collaboration with general physicians. What would these three priorities be?

Closure:

Is there anything else I haven't asked you that you would like to add?

- Thanking the participants again;
- Informing them that they will receive the summary, and later on, the results of the study;
- Reminding them that they can contact the first author at any time if they have questions or would like to withdraw their participation.
